# Strategy and suppression impairments after right lateral prefrontal and orbito-frontal lesions

**DOI:** 10.1093/brain/awv269

**Published:** 2015-09-16

**Authors:** Lisa Cipolotti, Colm Healy, Barbara Spanò, Francesca Lecce, Francesca Biondo, Gail Robinson, Edgar Chan, John Duncan, Tim Shallice, Marco Bozzali

**Affiliations:** ^1^ 1 Department of Neuropsychology, National Hospital for Neurology and Neurosurgery, London, UK; ^2^ 2 Dipartimento di Psicologia, Università di Palermo, Italy; ^3^ 3 Neuroimaging Laboratory, Santa Lucia Foundation, Rome, Italy; ^4^ 4 MRC Cognition and Brain Sciences Unit, Cambridge, UK; ^5^ 5 School of Psychology, The University of Queensland, Brisbane, Australia; ^6^ 6 Department of Experimental Psychology, University of Oxford, UK; ^7^ 7 Institute of Cognitive Neuroscience, University College London, UK; ^8^ 8 International School for Advanced Studies (SISSA-ISAS), Trieste, Italy

Sir,

We read with interest the scientific commentary by Hornberger and Bertoux (2015) on our study on the specificity of prefrontal cortex subregions for strategy use, verbal initiation and suppression (Robinson *et al.*, 2015). We administered Section 1 and 2 of the Hayling sentence completion task (Burgess and Shallice, 1997) to a large group of frontal and posterior patients. Section 1, assessing verbal initiation, requires the subject to complete sentences with an appropriate word (e.g. ‘*The captain stayed with the sinking*…’ could be completed by saying ‘*ship*’). Section 2, assessing inhibition/suppression, requires the completion of sentences with an unconnected word (e.g. ‘*London is a very busy*…’, could be completed by saying…‘*banana*’). This section also assesses the ability to adopt appropriate strategies. Healthy subjects are known to use heuristics in order to generate words unrelated to the sentence frame. Frontal patients may produce suppression errors (e.g. ‘*London is a very busy*…’ may be completed with ‘…*city*’). We found that right lateral (RL) patients were impaired on three critical variables, whereas patients with left lateral (LL) or superior medial lesions were not. Right lateral patients produced a significantly greater number of Suppression Errors, fewer Number of Correct Answers in Section 2 and had a larger Response Time difference (RTs Section 2 − RTs Section 1), a measure taken to indicate the additional ‘thinking time’ required to generate unconnected rather than appropriate words. We suggested that the right lateral region has a key role in generating or implementing an effective strategy. Other studies have previously documented that lesions in right rostral prefrontal cortex or right inferior frontal gyrus are linked to suppression impairments (Roca *et al.*, 2010). However, our study was the first to link these deficits to impairment in strategy use.

As noted by Hornberger and Bertoux (2015), failures of suppression and strategy use after right inferior frontal lesions may be linked to the idea that the inferior frontal cortex is involved in inhibition. In particular, right inferior frontal gyrus damage has been associated with inhibitory failures on stop-signal tasks (for a review see Aron, 2014). They further suggested that inhibitory deficits may also follow orbitofrontal lesions, questioning the specificity of our right lateral findings. As our previous study did not include orbitofrontal patients, here we present new data specifically comparing right lateral and orbitofrontal patients.

For research purposes 25 frontal patients (24 brain tumours; one stroke) and 28 healthy controls (HC) underwent cognitive investigation and assessment of their frontal lesions based on detailed anatomical localization using standard atlases (Duvernoy, 1991). Of note, all frontal lesions were entirely located within the frontal lobe and identified on T_1_-weighted images obtained by either 3 T (*n* = 10) or 1.5 T (*n* = 15) Siemens magnetic resonance scanners. Lesions were outlined by a neurologist (B.S.) blind to the experimental results, using a semi-automated local threshold contouring software (Jim 5.0, Xinapse System, http://www.xinapse.com/). A lesion mask was created for each patient by assigning a value of 1 to every voxel corresponding to a lesion and a value of 0 elsewhere. T_1_-weighted images were warped into the Montreal Neurological Institute space. The same transformation was applied to the corresponding lesion mask. Using this procedure we identified 11 patients with focal right lateral (*n* = 5) and orbitofrontal (*n* = 6) lesions. We calculated the percentage of volume of damage in either right lateral or orbitofrontal cortex (= right lateral lesion volume/total right lateral volume × 100). Using Kolmogorov-Smirnov Z, with exact probability statistics as an indicator of significance, we found no significant difference between right lateral and orbitofrontal patients in terms of percentage of lesion volume in either right lateral or orbitofrontal cortex (z = 0.716, *P* = 0.591). Figure 1 illustrates a probabilistic lesion map indicating the percentage of patients with a lesion in a given brain area for each group (i.e. right lateral; orbitofrontal)

**Figure 1:**
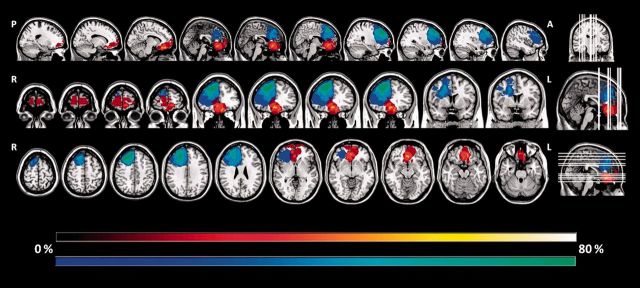
**Probabilistic lesion maps, indicating the percentage of patients with lesion in a given brain area, overlaid onto a T_1_-weighted image in Montreal Neurological Institute space.** Right lateral lesions are depicted in cool shades and orbitofrontal lesions in hot shades. A = anterior; L = left; P = posterior; R = right.

Healthy controls and frontal patients were matched for age [*t*(51) = −0.228, *P* = 0.820; mean age HC = 49.1, standard deviation (SD) = 15.8; patients = 48.1, SD = 15.5] and education [*t*(51) = −1.835, *P* = 0.072, mean years of education HC = 14.5, SD = 2.1; patients = 13.3, SD = 2.5]. There was no significant difference between right lateral and orbitofrontal patients in terms of age (t(9) = −0.507, p = 0.625), years of education [t(9) = −0.415, P = 0.688] or chronicity [*t*(9) − 0.507, *P* = 0.625; *t*(9) − 0.415, [z = 0.440, *P* = 0.883; mean days between lesion onset/tumour resection and assessment was 12.2 (SD = 12.55) and 17.83 (SD = 12.16) days for right lateral and orbitofrontal patients, respectively].

The Hayling task was administered according to published procedure (Burgess and Shallice, 1997). Suppression Errors, Number of Correct Answers in Section 2 and Response Time difference were calculated as detailed by Burgess and Shallice (1996). We also administered the Advanced Progressive Matrices (APM; Raven, 1976) to assess current level of non-verbal abstract reasoning and the Graded Naming Test (GNT; McKenna and Warrington, 1983) to assess nominal functions. Median scores and ranges for all tasks are shown in Table 1.

**Table 1:** Cognitive and Hayling scores

	HC *n* = 28	Frontal patients *n* = 25	RL *n* = 5	OF *n* = 6
**APM** (*n* correct)	10 (6–12)	7*** (1–11)	7 (2–9)	7 (1–10)
**GNT** (*n* correct)	24 (15–28)	**19** ** (8–26)	17 (12–23)	18 (8–26)
**Suppression Error scaled scores** **^a^**	6.5 (1–8)	2*** (1–7)	**1^#^** (1–7)	4.5 (2–7)
**Correct responses on Section 2, *n***	13 (5–15)	6*** (1–14)	**4^#^** (1–14)	9 (6–14)
**Response time difference (s)** **^b^**	14.5 (−14 to 63)	42*** (−7 to 164)	**42^#^** (38 to 70)	23.5 (−7 to 40)

Values are shown as median (range).

HC = healthy control; RL = right lateral; OF = orbitofrontal.

^**^ *P* < 0.01; ^***^P < 0.001 compared to HC; ^#^*P* < 0.05 compared to orbitofrontal.

^a^1–10 scaled score: 1 = out of the normal range; 2 = 1st percentile; 3 = 5th percentile; 4 = 10th percentile; 5 = 25th percentile, 6 = 50th percentile, 7 = 75th percentile, 8 = 90th percentile, 9 = 95th percentile and 10 = 99th percentile.

^b^Reaction Time Section 2 − Reaction Time Section 1.

Frontal patients performed significantly worse than healthy controls on the APM [*t*(40) = −3.774, *P* = 0.001] and GNT [*t*(24.07) = −2.981, *P* = 0.006]. There were no significant differences in the performance of the right lateral and orbitofrontal patients on either the APM (z = 0.516, *P* = 0.886) or GNT (z = 0.495, *P* = 0.896).

Compared to healthy controls, frontal patients were significantly impaired on: Suppression Errors (U = 99.00, z = −4.712, *P* < 0.001); Number of Correct Answers in Section 2 (U = 134.00, z = −3.75, *P* < 0.001 and Response Time difference (U = 99.00, z = −4.474, *P* < 0.001).

Right lateral patients made significantly more Suppression Errors than orbitofrontal patients (z = −1.321, *P* = 0.039)*.* All but one of the right lateral patients scored below the first percentile, indicating performance out of the normal range. In contrast, no orbitofrontal patient performed below the first percentile, and notably, two orbitofrontal patients obtained a score at the 75th percentile. We also found a significant difference between right lateral and orbitofrontal groups in the Number of Correct Answers in Section 2 (z = −1.321, *P* = 0.037). Four of five right lateral patients only produced four or fewer unconnected words (out of 15), which precluded a meaningful analysis of strategy use. In contrast, four of six orbitofrontal patients produced at least 9/15 correct answers, 18.3% of which fitted a standard strategy. Furthermore, right lateral patients had a significantly higher Response Time difference than orbitofrontal patients (z = −1.376, *P* = 0.026). Right lateral patients were almost twice as slow as orbitofrontal patients who performed more similarly to healthy controls.

Thus, our right lateral patients when faced with the task of rejecting an inappropriate prepotent response made many suppression errors. They also produced very few unconnected words and required longer *‘*thinking’ times, which are known to correlate with fewer strategy responses (Burgess and Shallice, 1996). This pattern of performance suggests strategy impairment. This is in accordance with studies documenting strategic failure following right-sided lesions in tasks requiring generation of an efficient multi-tasking strategy (Hotel Test, Roca *et al.*, 2010) or lateral lesions in semantic fluency tasks (Reverberi *et al.*, 2006).

The majority of our orbitofrontal patients obtained a suppression score in the normal range. They also produced a large number of correct responses on Section 2 and a speed of responding suggestive of strategy use. Thus, compared to right lateral cortex, orbitofrontal cortex seems less related to suppression/inhibition deficit in the Hayling Test.

Volle and colleagues (2012) found no significant difference in suppression errors between patients with lesions in posterior or rostral prefrontal cortex. However, a search for regions most associated with these errors suggested a focus in right Brodmann area 11. Interpretation of this result is limited since there was a degree of overlap between right lateral and orbitofrontal lesions and only two patients had lesions in the critical orbitofrontal area. Hornberger and colleagues (2011) reported impairment in suppression errors in 14 patients with frontotemporal dementia, which correlated with atrophy in ventro-medial orbito frontal cortex, subgenual as well as anterior temporal and medial frontal grey matter. This interesting finding may also warrant some caution given the somewhat limited localization value of neurodegenerative lesions.

In conclusion, our current findings corroborate the notion that the right lateral cortex is involved in strategy use. They provide preliminary results suggesting that orbitofrontal damage is less likely to give rise to suppression impairment on the Hayling task. Future lesion studies are needed to delineate further the functionality of the right lateral and orbitofrontal cortex in suppression/inhibition.

## Funding

This work was supported by the Wellcome Trust Grant (089231/A/09/Z). This work was undertaken at UCLH/UCL, which received a proportion of funding from the Department of Health’s National Institute for Health Research Biomedical Research Centre’s funding scheme.
